# A Scoping Review and Risk Assessment of Aflatoxin B1 Contamination in Rice, Maize, and Peanut-Based Products Across Selected ASEAN Countries

**DOI:** 10.3390/foods15111874

**Published:** 2026-05-25

**Authors:** Siti Soleha Ab Dullah, Nurul Izzah Ahmad, Nurfatehar Ramly, Norizzati Amsah, Sumarni Mohd Ghazali, Siti Hajar Muhamad Rosli, Hussin Muhammad

**Affiliations:** 1 Toxicology and Pharmacology Unit, Herbal Medicine Research Centre, Institute for Medical Research (IMR), National Institutes of Health, Persiaran Setia Murni, Setia Alam, Shah Alam 40170, Selangor, Malaysia; ssoleha@moh.gov.my; 2 Infectious Disease Research Centre, Institute for Medical Research (IMR), National Institutes of Health, Persiaran Setia Murni, Setia Alam, Shah Alam 40170, Selangor, Malaysia; nurulizzahmad69@gmail.com; 3 Centre for Infectious Disease Epidemiology Research, Institute for Public Health (IKU), National Institutes of Health, Persiaran Setia Murni, Setia Alam, Shah Alam 40170, Selangor, Malaysia; nurfatehar@moh.gov.my; 4 Centre for Family Health Research, Institute for Public Health (IKU), National Institutes of Health, Persiaran Setia Murni, Setia Alam, Shah Alam 40170, Selangor, Malaysia; norizzati.a@moh.gov.my; 5 Biomedical Epidemiology Unit, Special Resource Centre (SRC), Institute for Medical Research (IMR), National Institutes of Health, Persiaran Setia Murni, Setia Alam, Shah Alam 40170, Selangor, Malaysia; sumarni.mg@moh.gov.my; 6 Integrative Health Information Unit, Herbal Medicine Research Centre, Institute for Medical Research (IMR), National Institutes of Health, Persiaran Setia Murni, Setia Alam, Shah Alam 40170, Selangor, Malaysia; sitihajar.rosli@moh.gov.my

**Keywords:** aflatoxin B1, ASEAN, dietary exposure, margin of exposure, liver cancer risk, peanuts, maize, rice

## Abstract

Background: Aflatoxin B1 (AFB1) is a potent hepatocarcinogen commonly found in staple foods from tropical regions. Aim: This scoping review collated existing evidence on AFB1 contamination in rice, maize, and peanut-based products across ASEAN countries to estimate chronic dietary exposure, Margin of Exposure (MOE), and the associated liver cancer risk. Methods: A systematic search was performed in five databases. Estimated Daily Intake (EDI) and risk metrics were calculated using sample-size weighted mean concentrations, along with regional consumption data. Risk characterisation used the benchmark dose lower confidence limit (BMDL_10_) of 400 ng/kg BW/day, while liver cancer potency levels were adjusted according to Hepatitis B virus (HBV) prevalence for each population. Results: Twenty studies from Malaysia, Thailand, Vietnam, and the Philippines met the inclusion criteria. Peanuts and maize had the highest AFB1 concentrations among all food groups. Peanuts showed the highest contamination in the Philippines, followed by Malaysia, Vietnam, and Thailand. Maize exhibited a similar trend, with the highest levels observed in the Philippines. Most MOE values calculated were below 10,000, indicating a major public health concern. An exception was peanuts in Thailand, where MOE values exceeded 10,000, thus indicating a lower genotoxic carcinogenicity risk. The estimated liver cancer burden due to dietary AFB1 varied widely among countries and commodities. Conclusions: These findings show significant differences in AFB1 exposure in the ASEAN region. There is a need for improved surveillance, better post-harvest management, and harmonised regional risk management strategies.

## 1. Introduction

Aflatoxins (AFs) are a group of mycotoxins produced primarily by *Aspergillus flavus* and *Aspergillus parasiticus*, which commonly contaminate grains and nuts in tropical and subtropical regions [1]. The major types include aflatoxin B1 (AFB1), aflatoxin B2 (AFB2), aflatoxin G1 (AFG1), and aflatoxin G2 (AFG2), collectively referred to as total aflatoxins (TAF). In addition, hydroxylation of AFB1 and AFB2 results in the formation of aflatoxin M1 (AFM1) and aflatoxin M2 (AFM2), which are commonly detected in milk and dairy products [2]. Among these, AFB1 is the most potent and has been classified by the International Agency for Research on Cancer (IARC) as a Group 1 human carcinogen due to its strong genotoxic and hepatocarcinogenic properties [3,4].

The Association of Southeast Asian Nations (ASEAN) comprises eleven countries: Brunei Darussalam, Cambodia, Indonesia, Lao PDR, Malaysia, Myanmar, the Philippines, Singapore, Thailand, Vietnam, and Timor-Leste. These countries are characterised by tropical climatic conditions that favour the growth of *Aspergillus* species and increase the risk of aflatoxin contamination in food commodities. Across ASEAN, aflatoxin contamination has been widely reported in staple commodities such as maize and peanuts, often exceeding international safety limits [5,6]. Studies in Indonesia and Malaysia have identified *Aspergillus flavus* as the dominant aflatoxin-producing fungus, with a high prevalence of toxigenic isolates and reported contamination levels exceeding 2000 µg/kg in various agricultural commodities [7,8].

The widespread occurrence of aflatoxin contamination in staple foods highlights the need for comprehensive risk assessment to evaluate dietary exposure and associated health risks. As major components of daily diets, these commodities are key sources of AFB1 exposure. Health impacts include acute aflatoxicosis and chronic toxicity, particularly hepatocellular carcinoma (HCC) and childhood stunting [9,10]. The risk of HCC from aflatoxin exposure is significantly increased in the presence of chronic Hepatitis B virus (HBV) and Hepatitis C virus (HCV) infections due to their synergistic effects [11].

Risk assessment studies across ASEAN have consistently reported contamination in staple foods, with peanuts and maize frequently identified as high-risk commodities and associated with low margin-of-exposure (MOE) values. However, most existing studies are limited to individual countries and do not provide a comprehensive regional comparison integrating contamination data, dietary exposure, and risk characterisation across multiple food commodities. Therefore, this study aims to address this gap by conducting a regional risk assessment of AFB1 in peanuts and cereals across ASEAN countries and by providing a comparative evaluation of dietary exposure and associated health risks.

## 2. Materials and Methods

### 2.1. Literature Search

#### 2.1.1. Protocol and Registration

This scoping review was conducted in accordance with the PRISMA Extension for Scoping Reviews (PRISMA-ScR) guidelines. The protocol for this review was pre-registered on PROSPERO on 28 November 2025 (Registration ID: CRD420251240464).

#### 2.1.2. Literature Search and Search Strategy

The literature search was conducted in January 2025 using the following electronic databases: Scopus, Wiley Online Library, PubMed, Web of Science, and Embase. The search strategy was intentionally broad at the initial stage to maximise sensitivity and capture a wide range of potentially relevant studies. The main keywords searched were “aflatoxin” and the commodity names “rice”, “maize”, “corn”, and “nut” contained in the title and abstract. The detailed query search strategy for each database is provided in Supplementary Table S1. A publication year filter was applied to include studies published between 2010 and 2025. No additional restrictions were applied at this stage to avoid unnecessary exclusion of potentially relevant records.

#### 2.1.3. Study Selection

Identification of relevant studies was based on a pre-defined Population–Concept–Context (PCC) framework, as recommended for scoping reviews [12]. Following the removal of duplicate records, the remaining studies were screened based on titles and abstracts to exclude articles that were clearly irrelevant. Primary references of all included sources of evidence were hand-searched, screened, and included as additional studies where relevant. The study selection process was conducted by two independent authors in pairs. Any discrepancies during the study selection process were further discussed and resolved with the lead author to ensure consistency and transparency. The inclusion and exclusion criteria applied in this review are summarised in Table 1.

#### 2.1.4. Data Charting and Extraction

For each included study, the following information was extracted: country of study, reference details (author and year), food type, sample size (n), toxin type, and quantitative measures of aflatoxin contamination. Extracted concentration data included mean AFB1 levels (µg/kg), standard deviation (SD; µg/kg), minimum and maximum values (µg/kg), and median concentrations (µg/kg), where available. All extracted data were recorded in a standardised data extraction form to ensure consistency across studies. The data charting process was iterative, allowing refinement of extracted variables to accommodate variations in reporting formats across studies. When certain statistical parameters were not reported, the available data were extracted as presented without imputation to preserve the integrity of the original study findings.

#### 2.1.5. Data Synthesis

The reporting of results followed the PRISMA Extension for Scoping Reviews (PRISMA-ScR) guidelines to enhance transparency and reproducibility of the review process (Supplementary Table S2). The extracted data were collated and summarised descriptively to provide an overview of AFB1 contamination across food commodities and selected ASEAN countries. Findings were synthesised using tables and narrative summaries to highlight the distribution of AFB1 concentrations and study characteristics, and to identify research gaps.

### 2.2. Exposure Assessment in Peanuts and Peanut-Based Products, Rice and Rice-Based Products, and Maize and Maize-Based Products

#### 2.2.1. Aflatoxin B1 Occurrence and Summary Measures

For each eligible study, information on the reported mean AFB1 concentration (µg/kg), food type, number of sampling sites, and total number of analysed samples was systematically compiled. For studies reporting total aflatoxins (TAF), AFB1 concentrations were estimated using commodity-specific conversion factors adopted from the ARAC report published in 2020 [13]. This approach derives AFB1 from TAF based on country-specific data, particularly from Indonesia [14] and Malaysia [15,16]. The applied conversion factors are presented in Supplementary Table S3. Food items were categorised into three commodity groups to ensure consistency across studies. Specifically, peanut and peanut-based products were grouped under the peanut category; rice, rice-based products, and coloured rice were grouped under the rice category; and maize, corn, and maize-based products were combined into the maize category.

Pooled mean AFB1 concentrations were calculated using a sample-size weighted (SSW) mean approach, incorporating all studies that reported mean concentration and sample size, irrespective of whether a standard deviation (SD) was reported. This approach was selected to derive representative central estimates for dietary exposure and risk assessment, where population-level contaminant concentrations are the primary interest [17,18,19]. Weighted mean concentrations were calculated as the sum of the products of study-specific mean concentrations and sample sizes divided by the total number of samples within each country–commodity group. Studies that did not report a mean concentration were excluded from the analysis, including those presenting only minimum and maximum values, median values, or qualitative results (e.g., “not detected”, “positive”, or “<LOD”), in accordance with standard evidence synthesis guidance [17,20]. Entries without a reported sample size (n) or with non-numeric sample size values (e.g., “NR”, “–“) were also excluded. Although this approach may introduce selection bias, as excluding studies reporting non-mean values may limit the representation of variability in contamination levels and potentially lead to under- or overestimation of exposure, it was necessary to ensure consistency and comparability in exposure estimation. The resulting harmonised pooled concentration dataset served as the primary input for subsequent dietary exposure and margin of exposure (MOE) assessments for AFB1 [19]. Statistical analyses were performed using RStudio (R version 4.4.1).

#### 2.2.2. Estimated Daily Intake (EDI) of AFB1

The EDI of AFB1 for rice, maize, and peanuts was determined using country-specific food consumption rates and adult body weight (BW) assumptions, obtained from national surveys and published literature [21,22,23,24,25,26,27,28,29,30,31] (Supplementary Table S4). Commodity-specific EDIs of AFB1 were calculated separately for rice, maize, and peanuts using the standard exposure assessment (Equation (1)):

(1) $${\mathrm{EDI}}_{i}=\frac{C_{i}\times {\mathrm{CR}}_{i}}{\mathrm{BW}}$$

C_i_: represents the pooled mean concentration of AFB1 in commodity i (µg/kg).

CR_i_: the consumption rate of commodity i (kg/person/day).

BW: the mean adult BW (kg) for the country under assessment.

SSW mean concentrations were used as central estimates for dietary exposure assessment. To characterise uncertainty in dietary exposure estimates, low-, typical-, and high-concentration scenarios were defined for each country–commodity combination. The selection of concentration values for these scenarios was guided by the number of independent contributing studies (*k*). When sufficient data were available (*k* ≥ 3), uncertainty bounds were derived using non-parametric bootstrap resampling of study-level mean concentration estimates. The lower and upper bounds of the bootstrap distribution were used to define the low and high exposure scenarios, respectively, while the SSW mean was used as the typical exposure estimate. For datasets with limited data (*k* = 2), the minimum and maximum reported mean concentrations were used to define the low and high scenarios. When only a single study was available (*k* = 1), the reported mean concentration was applied to all exposure scenarios and flagged to indicate high uncertainty. These low- and high-exposure scenarios were used solely for uncertainty and sensitivity exploration in EDI estimation.

### 2.3. Margin of Exposure (MOE) Assessment Using the Benchmark Dose Approach for AFB1

The benchmark dose lower confidence limit corresponding to a 10% increase in cancer incidence (BMDL_10_) value of 0.4 µg/kg BW/day was adopted as recommended by EFSA [19]. The MOE was calculated as the ratio between the BMDL_10_ and the EDI (Equation (2)):

(2) $$\mathrm{MOE}=\frac{{\mathrm{BMDL}}_{10}}{\mathrm{EDI}}$$

BMDL_10_: benchmark dose lower confidence limit value of 0.4 µg/kg BW/day.

EDI: estimated daily intake of AFB1 (µg/kg BW/day).

In accordance with established risk assessment benchmarks, MOE values of 10,000 or greater from lifetime exposure (up to 75 years) are considered indicative of low concern for public health; and therefore, have low priority for risk management. The MOE values used for overall risk characterisation were derived from typical exposure estimates, which are considered most representative of population-level exposure [18,19,20,32].

### 2.4. The Percentage of Liver Cancer Attributable to AFB1 Contamination in Samples

The estimated liver cancer risk was calculated for each country by combining the total dietary exposure to AFB1 (following low, typical, and high exposure scenarios) from peanuts and peanut-based products, rice and rice-based products, and maize and maize-based products with the population-specific average potency (Equation (3)). The average potency was calculated by weighting AFB1 carcinogenic potency by hepatitis B surface antigen (HBsAg) status.

(3) $${\mathrm{LCR}}^{\mathrm{country}}=\mathrm{Total}\;{\mathrm{EDI}}^{\mathrm{scenario}}\times \mathrm{Average}\;\mathrm{Potency}$$

LCR^country^: Liver cancer risk per 100,000 population (according to country).

Total EDI^scenario^: Cumulative EDI of AFB1 from all commodities following low, typical or high scenarios.

Potency values of 0.01 and 0.3 liver cancer cases per year per 100,000 population per ng/kg BW/day were applied to HBsAg-negative and HBsAg-positive individuals, respectively [33]. These values represent global estimates established by FAO/WHO JECFA and were weighted using country-specific prevalences of HBsAg positivity, which were reported to be 1.6% for Malaysia [34], 0.4% for Thailand [35], and 16.7% for the Philippines [36]. For Vietnam, the prevalence of HBsAg positivity in the adult population was obtained from nationally reported survey data, with an estimated prevalence of 7.2%, as reported by the WHO Global Health Observatory [37].

The proportion of liver cancer cases attributable to dietary aflatoxin exposure was subsequently estimated as the ratio of the calculated population risk to the age-standardised liver cancer incidence rate for Asia (Equation (4)):

(4) $$\%\;{\mathrm{LCR}}^{\mathrm{AF}}=\frac{\mathrm{Risk}\;\mathrm{scenario}}{\mathrm{INC}}\times 100$$

% LCR^AF^: Estimated percentage of liver cancer attributable to dietary AFB1 exposure.

INC: The age-standardised liver cancer incidence rate for Asia (11.6 per 100,000 population) [38].

## 3. Results

### 3.1. Literature Search and Study Selection

**Figure 1:**
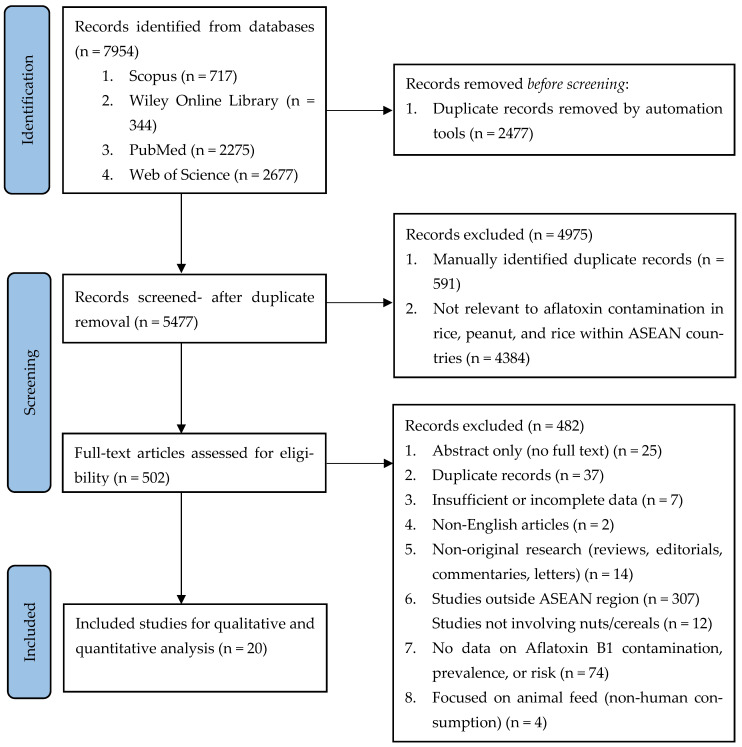
PRISMA flow diagram of literature search.

The study selection process is illustrated in the PRISMA flow diagram (Figure 1). A total of 7954 records were initially identified from the selected databases, including one additional record identified through manual reference list screening. After automatic duplicate removal using EndNote (n = 2477), 5477 records remained for title and abstract screening. At this stage, 4975 records were excluded because they were not relevant to AFB1 contamination in the selected food commodities, were conducted outside the selected ASEAN countries, or did not meet the study scope. Consequently, 502 full-text articles were assessed for eligibility. Of these, 482 articles were excluded for reasons including insufficient quantitative data, studies conducted outside the selected ASEAN countries, non-original articles, or studies unrelated to the selected commodities. Twenty studies from four selected ASEAN countries (Malaysia, Thailand, Vietnam, and the Philippines) met the predefined eligibility criteria and were included in this review. Of these, studies that met the criteria for risk assessment were subsequently used for pooled concentration estimation and dietary risk assessment.

### 3.2. Included Studies’ Characteristics

**Figure 2:**
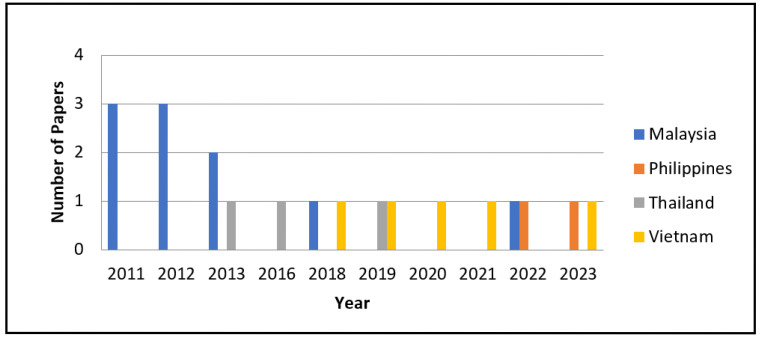
Number of included studies by country of publication.

Figure 2 presents the temporal distribution of the 20 studies included in this review across four selected ASEAN countries between 2011 and 2023. Malaysia contributed the highest number of studies (n = 10), with publications concentrated mainly in the earlier years, particularly in 2011 and 2012. Vietnam contributed five studies, with publications reported more consistently from 2018 onwards. Thailand contributed three studies, which were distributed intermittently across the study period. The Philippines contributed the fewest studies (n = 2), with publications reported only in the most recent years (2022–2023).

Table 2 summarises the characteristics and main findings of the 20 studies included in this review on aflatoxin contamination in nuts and cereals across selected ASEAN countries. The studies were conducted between 2003 and 2023 and covered Malaysia, Thailand, Vietnam, and the Philippines, with Malaysia contributing the highest number of studies. Sampling was carried out at various points along the food supply chain, including retail markets, supermarkets, farmer cooperatives, manufacturers, importers, and household or community settings. Most studies focused on commonly consumed food commodities, particularly peanuts, rice, maize, and cereal-based products, with sample sizes ranging from fewer than 50 to over 1000 samples. AFB1 is the most frequently reported toxin across all studies, although several investigations also measured other aflatoxins (AFB2, AFG1, and AFG2). Analytical methods commonly employed included HPLC, LC–MS/MS, UPLC-FLD, and ELISA, with chromatographic techniques being the most widely used.

Aflatoxin contamination was commonly detected, with considerable variation in prevalence and concentration across countries and food types. Overall, higher contamination levels were generally found in peanuts and maize. Severe peanut contamination was particularly reported in the Philippines by Rustia et al. [22], while elevated maize contamination was observed in Vietnam [39,40], with several samples exceeding national regulatory thresholds. In contrast, studies from Malaysia and Thailand generally reported lower aflatoxin concentrations in rice and cereal products [41,42], although some samples, particularly peanuts, still exceeded regulatory limits [43,44].

**Table 2:** Characteristics of the studies and highlights on findings of Aflatoxin analysis from ASEAN countries.

Study	Country	Study Aims	Sampling Date	Study Locations	Sample Types, (N)	Analyte/Methods	Findings
[43]	Malaysia	To determine the levels and critical points of aflatoxins and fungal contamination in peanuts along the supply chain	November 2014 to February 2015	Samples were collected across Malaysia from importers, manufacturers, and retailers.	Total N = 178: raw peanuts (n = 87) and peanut-based products (n = 91)	AFB1, AFB2, AFG1 and AFG2/HLPC	Aflatoxin contamination was higher in raw peanuts and peanut-based products from retailers compared to manufacturers. Raw peanut kernels from retailers showed the highest levels (mean: 120.7 µg/kg), with 38% of samples exceeding Malaysian limits, versus 22% from manufacturers (mean: 20.5 µg/kg). No aflatoxins were detected in importer samples. For peanut-based products, 15.0% of retailer samples and 5.9% of manufacturer samples exceeded the permissible limits.
[45]	Malaysia	To determine the occurrence of mycotoxigenic fungi and mycotoxin contamination on red rice at the consumer level in Selangor, Malaysia.	NA	Samples were collected from traditional Chinese medicine shops across 9 districts in Selangor, Malaysia.	Total N = 50 (red rice)	Citrinin, TAF and Ochratoxin-A/ELISA analysis	Aflatoxins were detected in 92% of red rice samples (0.61 to 77.33 μg/kg; mean:14.72 ± 16.24 μg/kg). Overall, 70% exceeded the Malaysian limit (5 μg/kg) and the European Commission limit (4 μg/kg), indicating high contamination levels in the tested red rice samples.
[31]	Malaysia	To determine the exposure of the adult Malaysian population to aflatoxins through the diet; To identify foods that contribute significantly to the intake of aflatoxins; To estimate liver cancer risk of the adult population attributable to dietary intake of aflatoxins.	2003	Food samples were collected across six regions of Malaysia (North, Central, South, East, Sabah, and Sarawak) from retail markets.	Total N = 236 (from 38 food items)	AFB1 and TAF/HPLC	AFB1 was detected in 4.2% of samples, mainly in peanuts, peanut butter, and breakfast cereals. The highest level was 117.3 µg/kg, with total aflatoxins reaching 140.9 µg/kg in peanuts. Dietary exposure ranged from 0.47 to 34.00 ng/kg BW/day, with peanuts contributing over 80%. Estimated liver cancer risk ranged from 0.01 to 0.85 cases per 100,000/year, accounting for 0.2–17.3% of cases in Malaysia.
[46]	Malaysia	To develop an LC–MS/MS method for the simultaneous determination of aflatoxins, OTA, ZEA, DON, FB1, FB2, T2 and HT2-toxin.	May 2010 to June 2010.	Cereal samples were collected from general markets in Kuala Lumpur, Malaysia	Total N = 100: rice (n = 50), wheat (n = 20), oat (n = 10), barley (n = 10), and maize (n = 10)	TAF, OTA, ZEA, DON, FB1, FB2, T2 and HT2-toxin/LC-MS/MS	Aflatoxins were detected in 60% of cereal samples, most commonly in rice, followed by wheat, maize meal, barley, and oat. Concentrations ranged from 0.12 to 4.54 µg/kg, with only two rice samples exceeding the EU limit (4 µg/kg). Overall, contamination was widespread but generally low and within permissible limits.
[42]	Malaysia	To develop an FLD- and DAD-HPLC method with a chemical and photochemical post-column derivatisation system for the simultaneous determination of 12 mycotoxins; To assess a new AOFZDT multi-functional IAC for clean co-extraction of all toxins; To test method versatility via analysis of 45 cereal samples from the Malaysian market.	September 2009 to December 2009	Samples were collected from various markets in Kuala Lumpur, Malaysia	Total N = 45: rice (n = 30), wheat (n = 10), and maize flakes (n = 5)	12 mycotoxins/RP-HPLC	Aflatoxins were detected in 33.3% of rice, 30% of wheat, and 20% of maize samples. The concentrations ranged from 0.19 to 3.96 µg/kg, with mean levels of 2.12 µg/kg (rice), 3.16 µg/kg (wheat), and 0.25 µg/kg (maize). None exceeded the EU limit (4 µg/kg), indicating low contamination and minimal risk.
[47]	Malaysia	To develop an LC-ESI-CID-MS/MS analysis using a hybrid QTOF-instrument for the rapid determination of aflatoxin B1, B2, G1 and G2 in food.	December 2009	Samples were randomly obtained from groceries and stores in Kuala Lumpur, Malaysia.	Total N = 44: barley (n = 10), wheat (n = 10), soybean (n = 10), corn (n = 5), peanuts (n = 5), and peanut butter (n = 4)	AFB1, AFB2, AFG1 and AFG2/LC–ESI-QTOF-MS/MS	Aflatoxins were detected in several samples, with one peanut sample exceeding the EU limit (AFB1: 23.0 µg/kg; AFB2: 4.1 µg/kg). All other samples were within permissible levels, indicating generally low contamination and effective food safety control.
[48]	Malaysia	To develop and validate a simple, rapid and reliable HPLC method for the simultaneous determination of aflatoxins (B1, B2, G1 and G2) in food and feed samples; To determine aflatoxins in peanuts, rice and chilli samples collected from Penang, Malaysia.	NA	Samples were collected randomly from supermarkets and night markets in Penang, Malaysia	Total N = 24: rice (n = 5), peanuts (n = 9), and chilli (n = 10)	AFB1, AFB2, AFG1 and AFG2/HLPC	Aflatoxins were detected in 71% of samples, with total levels ranging from 1.10 to 44.20 µg/kg. Contamination was mainly driven by AFB1, followed by AFG1, with AFG2 also commonly detected. AFB1 levels exceeded the EU limit (2 µg/kg) in all samples, while total aflatoxins exceeded the limit (4 µg/kg) in rice (3/4), peanuts (2/4), and chilli (3/9) samples.
[8]	Malaysia	To investigate the incidence of *Aspergillus* spp. in food products; To evaluate the ability of *A. flavus* to produce aflatoxins; To determine the natural occurrence of AFB1 in foods marketed in Penang, Malaysia.	NA	Samples were collected from different supermarkets in Penang, Malaysia	Total N = 95: rice-based foods (n = 13), wheat-based foods (n = 14), corn-based foods (n = 8), oat-based foods (n = 10), peanuts (n = 13), sunflower (n = 7), sesame (n = 8), nuts (n = 7), chilli (n = 8), pepper (n = 4), and cumin (n = 3)	AFB1/ELISA	AFB1 was detected in 72.6% of samples (0.54–15.33 µg/kg, mean 1.95 µg/kg). Peanut products showed the highest contamination (1.47–15.33 µg/kg). All levels were below Malaysian limits (<35 µg/kg). The findings demonstrated widespread low-level contamination in nuts and foods from Malaysia.
[49]	Malaysia	To determine occurrence of AFB1 in nuts/nut products and assess dietary exposure and risk for Penang population	June 2008 to December 2008	Samples were collected from various retail outlets, markets, and food premises in Penang, Malaysia.	Total N = 128: nut and nut product samples	AFB1/LC–MS/MS	AFB1 was detected in 57% of samples (0.40–222 μg/kg), with 13.3% exceeding the EU limit (2 μg/kg). The highest mean level was observed in fried peanuts (58.9 μg/kg). Dietary exposure ranged from 0.36 to 8.89 ng/kg BW/day, with MOE values of 34–847 (<10,000), indicating a potential health concern. The estimated liver cancer risk was 0.03–0.73 cases per 100,000/year.
[16]	Malaysia	To determine the occurrence of aflatoxins (B1, B2, G1 and G2) in selected rice production areas in Malaysia.	March–June 2022	Samples collected from 36 BERNAS rice production areas across Peninsular and East Malaysia.	Total N = 36 (rice)	AFB1, AFB2, AFG1, AFG2/HPLC	Aflatoxins were detected in 36.11% of areas; total aflatoxin levels ranged from 0.73 to 7.61 µg/kg. Three sites exceeded the total aflatoxin level set in the EU and Malaysian limits (Kuching, KBB Sungai Limau, KBB Paya Keladi).
[50]	Philippines	To establish the profile of the potential risks associated with the consumption of peanuts contaminated with aflatoxin to the Filipino consuming population.	November 2019	Samples were collected from 5 major peanut-producing provinces in the Philippines (Ilocos Sur, Ilocos Norte, La Union, Pangasinan, and Isabela)	Total N = 50 (dried, shelled peanuts)	AFB1, AFB2, AFG1 and AFG2/LC-MS/MS	Aflatoxins were detected in 92% of peanut samples (0.51–2736.24 µg/kg; mean: 802.83 µg/kg), with AFB1 as the dominant toxin (mean: 683.53 µg/kg), followed by AFB2 (119.30 µg/kg). AFG1 and AFG2 were not detected. All samples exceeded the Codex limit (15 µg/kg), indicating severe contamination and potential public health risk.
[51]	Philippines	To determine the occurrence of AF and mycotoxigenic *Aspergillus* spp. in maize varieties in the region	Mac 2019 to April 2019	Samples were collected from 6 municipalities in Isabela, including the major producers Ilagan City, Cauayan City, Echague, and San Mariano, as well as the minor producers San Pablo and Aurora.	Total N = 107: hybrids (n = 101) and open-pollinated varieties (n = 6)	TAF/ELISA	Aflatoxin was found in half (50.5%) of the maize samples, with nearly a quarter (22.8%) exceeding the Philippine National Standard. Most samples complied with food (<20 μg/kg) and feed (<50 μg/kg) safety limits.
[52]	Vietnam	To evaluate the impact of the crop season, cultivation region, and traditional pre- and post-harvest agricultural practices on mycotoxin contamination in the Mekong Delta rice chain of Vietnam.	December 2017 to December 2019	Samples were collected from farmers and rice companies or cooperatives involved in contract farming in three regions: Can Tho, An Giang, and Dong Thap.	Total N = 230: paddy (n = 184) and white rice (n = 46)	23 mycotoxins/LC-MS/MS	Out of 230 rice samples, 45% were contaminated with mycotoxins, with aflatoxins among the most prevalent. Aflatoxins were detected in 50% of paddy and 20% of white rice samples. Three paddy samples exceeded the regulatory limit (5 µg/kg). Total aflatoxin levels ranged from 2.3 to 547 µg/kg.
[27]	Vietnam	To estimate the dietary exposure and health risk caused by mycotoxins for children under 5 years living in the Lao Cai province in Northern Vietnam.	NA	Samples were collected from Lao Cai province, which consists of nine sub-regions: Lao Cai city and eight other districts.	Total N =1080 (from 40 food items)	AFB1, Ochratoxin A and fumonisins/ELISA	AFB1 was detected in 87.5% of samples in Lao Cai, Vietnam, with the highest levels in egg and milk products, followed by oily seeds, meat products, and rice. Mean dietary exposure among children under five was 118.7 ng/kg BW/day, with rice contributing 52.2 ng/kg BW/day. The estimated cancer risk was 12.1 cases per 100,000/year, with a low MOE of 1.4, indicating a significant public health concern.
[40]	Vietnam	To assess the exposure to four mycotoxins based on the analysis of rice, maize, peanut and sesame samples in three provinces in Northern Vietnam and the food consumption data for 4 different age groups; To characterise the health risk of these mycotoxins from different regions.	October 2016 to September 2017	Samples were collected from three provinces in Northern Vietnam (Hanoi, Thanh Hoa, and Ha Giang)	Total N = 606: rice (n = 144), maize (n = 189), peanuts (n = 144), and sesame (n = 129)	AFB1, FB1, OTA, ZEA/ELISA	AFB1 was detected in 19.1% of samples, most commonly in maize, followed by peanuts, rice, and sesame. Mean levels ranged from 2.56 to 2.62 µg/kg (Hanoi), 5.31–5.39 µg/kg (Thanh Hoa), and up to 66.0–66.1 µg/kg (Ha Giang), exceeding the regulatory limit (5 µg/kg) in several maize and peanut samples. Estimated liver cancer risk ranged from 0.23 to 0.65 cases per 100,000/year in Hanoi and Thanh Hoa, increasing to 21.0 in Ha Giang.
[39]	Vietnam	To assess the levels of AFB1 in maize during the rainy season and in-depth knowledge/behaviour of aflatoxins among people in Son La province.	September 2016	Maize samples were collected in Son La Province, Vietnam, which includes five districts: Mai Son, Moc Chau, Son La City, Thuan Chau, and Yen Chau	Total N = 378 (maize)	AFB1/ELISA	A total of 378 maize samples were analysed. AFB1 contamination exceeded 5 µg/kg in 54.0% of samples and 20 µg/kg in 37.3%. Concentrations ranged up to 417.0 µg/kg (mean: 37.3 µg/kg; median: 19.4 µg/kg). The highest levels were observed in Mai Son District, followed by Son La City and Thuan Chau, with the lowest in Yen Chau.
[53]	Vietnam	To develop and validate a high-performance analytical method for the quantitation of aflatoxins B1, B2, G1, and G2 in peanut and raisin matrices, and to apply the validated method for the survey of aflatoxin contamination in related products collected in Ho Chi Minh City.	NA	Samples were collected from markets in the central districts of Ho Chi Minh City, Vietnam,	Total N = 700: peanuts (n = 350) and raisins (n = 350)	AFB1, AFB2, AFG1 and AFG2/UPLC-FLD	AFB1 was detected in 28.6% of peanut samples (0.31–554 µg/kg), with lower detection of AFB2 (13.4%), AFG1 (5.7%), and AFG2 (0.6%). Overall, 12.8% exceeded total aflatoxin limits and 13.4% exceeded the AFB1 limit. In contrast, only 0.8% of raisin samples contained trace AFB1 (0.82–1.48 µg/kg), all below permissible limits, indicating minimal contamination.
[54]	Thailand	To assess the intake of AFB1 by Thai population through consumption of contaminated brown and colour rice.	June/July 2012 and Dec 2012/Jan 2013	Samples were obtained from local market, retail shops and shops of farmer cooperative groups from 20 provinces of the central and northeast region of Thailand	Total N = 240: brown rice (n = 120) and coloured rice (n = 120)	AFB1/HPLC	AFB1 contamination was higher in period I (59%) than in period II (10%), with one sample exceeding the Thai limit and 5% surpassing the stricter EU limit. Estimated daily intake was higher in period I (800 ng/kg BW/day) than in period II (120 ng/kg BW/day). The projected cancer risk remained low at 0.011 cases per 100,000 persons per year.
[41]	Thailand	To determine the occurrence of multiple mycotoxins in barley and nine types of rice sold in Thailand and to assess consumer health risk.	April 2017 to July 2017	Samples were collected from supermarkets and retail shops in Bangkok, Thailand	Total N = 300: black sticky rice, brown rice, GABA rice, Japanese rice, jasmine rice, red rice, riceberry rice, white rice, white sticky rice, and barley (n = 30 each).	16 mycotoxins/LC-MS/MS	AFB1 was detected in several rice types, including GABA brown rice (6.67%), black sticky rice (20%), and riceberry rice (10%), while AFG1 was found in 3.33% of jasmine and white sticky rice samples. Mean AFB1 levels ranged from 1.38 to 1.48 µg/kg, all below EU limits (2 µg/kg for AFB1; 4 µg/kg total). AFB2 and AFG2 were not detected. Overall, contamination was low, with minimal health risk and an estimated cancer risk of 1 case per 1,000,000 population per year.
[55]	Thailand	To investigate contamination levels of aflatoxins in selected Thai commodities using validated HPLC methodology	NA	Samples were randomly collected from retail fresh markets and modern trade department stores in the central region of Thailand	Total N = 120: unpolished rice (n = 25), unpolished glutinous rice (n = 10), chilli powder (n = 30), whole dried chilli pods (n = 30), and raw peanuts (n = 25)	AFB1, AFB2, AFG1 and AFG2/HLPC	Aflatoxin contamination was detected across Thai commodities, highest in chilli powder (97%), followed by chilli pods (37%), peanuts (30%), glutinous rice (20%), and unpolished rice (4%). Mean total aflatoxin levels ranged from 0.16 to 25.43 µg/kg, with maximum levels up to 58.3 µg/kg. AFB1 was the predominant toxin, while AFG2 was not detected. Chilli powder showed the highest contamination severity, with 16 of 30 samples exceeding EU limits, whereas only one peanut and one glutinous rice sample exceeded the permissible levels.

NA: Not available.

### 3.3. Aflatoxin B1 Concentrations and Uncertainty Across Countries and Food Commodities

Table 3 summarises the SSW mean concentrations of AFB1 by country and food commodity, together with the associated uncertainty ranges. The number of contributing studies (k) varied across country–commodity combinations, ranging from one to four studies, with total sample sizes between 13 and 567. Peanuts exhibited the highest contamination levels overall, particularly in the Philippines (2877.26 µg/kg), followed by Malaysia (56.88 µg/kg) and Vietnam (30.81 µg/kg), while Thailand reported the lowest level (1.09 µg/kg). Maize showed moderate contamination, with the highest level observed in the Philippines (489.99 µg/kg), followed by Vietnam (140.30 µg/kg), and substantially lower levels in Malaysia (2.46 µg/kg). In contrast, rice consistently demonstrated lower AFB1 concentrations across all countries, with values generally below 6 µg/kg. Overall, peanuts had the highest AFB1 contamination levels, followed by maize, while rice showed comparatively lower levels.

### 3.4. Dietary Exposure and Risk Characterisation of Aflatoxin B1

Table 4 presents the estimated dietary exposure to AFB1 and the corresponding MOE for selected food commodities across the studied countries. Under the typical exposure scenario, dietary exposure varied substantially across countries and commodities. The highest exposure levels were observed for peanuts and maize in the Philippines (1392.96 and 318.88 ng/kg BW/day, respectively), indicating a major contribution to overall aflatoxin intake. In Vietnam, maize was the primary contributor (63.54 ng/kg BW/day), whereas in Malaysia, exposure was mainly driven by peanuts (51.66 ng/kg BW/day). In contrast, Thailand showed comparatively lower exposure levels, with rice as the main contributor.

Consistent with these patterns, MOE values indicated considerable variation in potential health risk. The lowest MOE values were observed for peanuts and maize in the Philippines (0.29 and 1.25, respectively), followed by maize in Vietnam (6.30) and peanuts in Malaysia (7.74). In comparison, higher MOE values were observed for maize in Malaysia (289.40) and rice in Vietnam (35.47), reflecting comparatively lower risk. Overall, most MOE values were below 10,000, indicating potential health concerns, particularly for peanuts and maize in high-exposure settings. The low and high-exposure scenarios showed consistent trends, with peanuts and maize remaining the primary contributors to exposure and risk across countries.

### 3.5. Liver Cancer Risk Estimates Attributable to Dietary Aflatoxin B1 Exposure Across ASEAN Countries

Table 5 summarises country- and commodity-specific estimates of liver cancer risk and attributable proportion under low, typical, and high AFB1 exposure scenarios, based on dietary intake and population-specific potency values. Estimated liver cancer risk varied markedly across countries and food commodities under the typical exposure scenario (Table 4). In Malaysia, the estimated liver cancer risk was primarily associated with peanut consumption, which accounted for the largest proportion of total dietary aflatoxin-related risk under the typical scenario (67.31%). Rice represented a substantial secondary source of risk (30.89%), reflecting high consumption levels despite lower AFB1 concentrations, whereas maize contributed minimally (1.80%). These findings indicate that peanuts were the dominant determinant of aflatoxin-related liver cancer risk in the Malaysian adult population.

In the Philippines, liver cancer risk under the typical exposure scenario was primarily driven by peanut consumption, which accounted for approximately 81.37% of the total estimated risk, while maize accounted for the remaining 18.63%. This pattern was consistent with the extremely high AFB1 concentrations reported in peanuts, resulting in markedly elevated estimated daily intakes and correspondingly low margins of exposure. In Thailand, the estimated liver cancer risk was almost exclusively attributable to rice consumption, which accounted for 99.81% of the total risk under the typical scenario. Despite moderate AFB1 concentrations, the high dietary intake of rice resulted in a disproportionately greater contribution to population-level risk, whereas peanuts contributed negligibly due to very low estimated exposure.

In Vietnam, maize was the principal source of liver cancer risk under the typical exposure scenario, accounting for approximately 75.46% of the total estimated risk. Rice and peanuts contributed smaller proportions, accounting for 13.39% and 11.15% of the total risk, respectively. This distribution reflects both elevated AFB1 concentrations in maize and significant dietary intake, identifying maize as the primary driver of aflatoxin-related liver cancer risk in the Vietnamese adult population. Across all countries, observed differences in liver cancer risk under the typical exposure scenario were determined by the combined effects of food-specific AFB1 contamination levels, consumption patterns, and country-specific average potency values that incorporate HBV prevalence.

## 4. Discussion

### 4.1. AFB1 Contamination Patterns Across Key ASEAN Commodities

Between 2011 and 2023, 20 studies investigating AFB1 in maize, rice, and peanuts across the ASEAN region were identified. Across these studies, a clear difference in contamination patterns was observed between food commodities. Peanuts and maize consistently exhibited higher AFB1 contamination levels compared to rice, reflecting the inherent susceptibility of oilseeds and maize to fungal invasion. The high lipid and nutrient content of peanuts and maize facilitates *Aspergillus* colonisation and subsequent mycotoxin biosynthesis during storage [46,56].

In tropical climates, delayed post-harvest processing, high moisture content, inadequate ventilation, and insect or mechanical damage can further promote *Aspergillus* growth and aflatoxin biosynthesis [47]. These findings align with observations in other tropical regions, such as Uganda, where high aflatoxin levels are strongly correlated with humid environments and suboptimal post-harvest handling [48]. In contrast, rice generally undergoes rigorous milling and more controlled storage conditions, which may reduce fungal proliferation and toxin accumulation, although its high dietary intake still renders it an important contributor to overall exposure [49,50].

### 4.2. Risk Characterisation from Dietary Exposure to AFB1

The observed variation in MOE values across the selected countries highlights a heterogeneous public health landscape. Critical MOE values (e.g., as low as 0.29 for peanuts in the Philippines) indicate limited safety margins between dietary exposure and toxicological reference points, suggesting elevated long-term health concerns. These findings demonstrate that AFB1-related risk is a multi-faceted interaction between contamination levels, dietary patterns, and population-specific vulnerabilities, notably Hepatitis B virus (HBV) prevalence.

The synergistic interaction between chronic AFB1 exposure and HBV infection may further amplify HCC risk. AFB1 induces DNA adduct formation and mutational damage, particularly in the TP53 tumour suppressor gene, while chronic HBV infection promotes sustained hepatic inflammation and impaired cellular repair mechanisms. Together, these effects substantially increase liver cancer susceptibility compared with either risk factor alone [51]. However, it is important to note that the MOE values reported in this study should be interpreted as conservative estimates of risk, ensuring that the risk characterisation prioritises a high level of consumer protection, particularly for populations with high rice and peanut intake, where exposure may be chronic.

Commodities that combine high contamination with high consumption, such as peanuts in the Philippines and maize in Vietnam, are key drivers of population-level risk. Differences in risk profiles across countries may also reflect variations in regulatory maximum levels (MLs), surveillance systems, and enforcement effectiveness. Countries with less consistent monitoring and post-harvest control practices may experience greater persistence of aflatoxin contamination within the food supply chain [47,52]. In addition, differences in storage infrastructure, regulatory implementation, and routine screening capacity may further contribute to variability in exposure risk across countries [53]. The dominance of peanuts as a risk contributor in the Philippines and Malaysia, and maize in Vietnam, reflects both elevated contamination levels and dietary relevance. In Thailand, rice accounted for the majority of the estimated risk due to high consumption, despite comparatively moderate contamination levels. These findings highlight the importance of considering both food contamination profiles and dietary patterns when prioritising risk management strategies.

### 4.3. Strengths and Limitations

This study builds upon the 2020 ASEAN Risk Assessment Centre for Food Safety (ARAC) [13] report by providing a more comprehensive risk characterisation. By systematically integrating data from four countries and applying a harmonised analytical framework, including SSW mean concentrations and scenario-based exposure modelling, this review offers a more detailed evaluation of regional dietary risk. However, the absence of eligible quantitative data from Indonesia, a major regional producer and consumer, means these findings may not fully reflect the entire ASEAN region.

Several limitations must be acknowledged. First, data were available from only four ASEAN countries, with no eligible quantitative studies from Indonesia, Cambodia, Lao PDR, Myanmar, Brunei Darussalam, Singapore, or Timor-Leste within the defined timeframe. Second, variability in sampling strategies, analytical methods, and reporting formats limited direct comparability across studies. Third, uncertainty estimation was constrained for country–commodity combinations with fewer than three studies (k < 3). In addition, the use of fixed conversion factors to estimate AFB1 from total aflatoxins (TAF) may introduce uncertainty into the exposure assessment, as the relative proportion of AFB1 can vary across commodities, fungal strains, and environmental conditions. Consequently, the estimated EDI, MOE, and cancer risk values may be influenced by variability in the applied conversion factors, particularly for country–commodity combinations with MOE values close to the reference threshold. Therefore, the derived estimates should be interpreted with caution.

Additionally, the exclusion of studies that did not report mean AFB1 concentrations or sample sizes, including those reporting only medians, ranges, or qualitative findings (e.g., “not detected” or “<LOD”), may have introduced selection bias. Studies with low, highly variable, or predominantly non-detectable contamination levels may have been more likely to use alternative summary measures rather than arithmetic means. Thus, the pooled concentration estimates may not fully reflect the entire distribution of reported contamination levels across ASEAN countries, potentially resulting in under- or overestimation of dietary exposure. Nevertheless, restricting inclusion to studies reporting quantitative mean concentrations and sample sizes was necessary to ensure methodological consistency and comparability for pooled exposure estimation. Despite these limitations, this review provides a structured regional overview using harmonised concentration estimates and consistent exposure modelling.

### 4.4. Future Directions and Recommendations

A critical evaluation of current regulatory frameworks across selected ASEAN member states reveals a significant lack of harmonisation in maximum levels (MLs) for aflatoxins, complicating both regional trade and public health protection. While some nations, such as Singapore and Malaysia, maintain stringent limits by aligning with or exceeding Codex Alimentarius standards, others have broader thresholds or lack specific MLs for processed rice and peanut derivatives. This regulatory heterogeneity creates loopholes where commodities exceeding regulatory limits in one market may potentially be redirected to markets with more permissive standards within the region. Furthermore, regional surveillance remains largely reactive rather than proactive monitoring, relying on localised academic surveys rather than systematic national longitudinal monitoring.

Seasonal surges in aflatoxin levels driven by the tropical ASEAN climate and increasingly volatile monsoon patterns often go undetected until the contaminated products reach end consumers. Pre-harvest initiatives such as integrating regional scaling of biological control using atoxigenic strains of *Aspergillus flavus* may be of substantial benefit, as these have demonstrated the ability to competitively displace toxigenic varieties and reduce aflatoxin levels by 70% to 95% [54]. Identification of contamination hotspots can also be strengthened through AI-driven predictive modelling for the synthesis of real-time weather data and satellite imagery [55] as a means of climate-resilient preventive agriculture. For post-harvest and processing stages, the adoption of novel non-thermal technologies offers a sustainable path to lowering the high dietary exposure identified in this review. Atmospheric Cold Plasma (ACP) has emerged as a particularly promising tool for ASEAN staples like rice and peanuts, capable of degrading AFB1 by over 80% while maintaining the sensory and nutritional integrity of the food [57].

To mitigate the low MOE values identified in this study, ASEAN member countries must shift towards a harmonised, risk-based surveillance system. Such a system should prioritise high-consumption staples like rice and implement standardised sampling protocols to ensure that data generated in one member state is comparable and actionable across the entire region. Strengthening the ASEAN Rapid Alert System for Food and Feed (ARASFF) by integrating real-time aflatoxin occurrence data would be a vital step towards a unified regional defence against chronic dietary exposure and the associated health risks.

## 5. Conclusions

This review highlights substantial variation in AFB1 contamination and associated dietary risk across selected ASEAN countries, with peanuts and maize identified as key contributors. The consistently low MOE values observed for several country–commodity combinations indicate a potential public health concern. These findings emphasise the importance of targeted, commodity-specific risk management strategies that consider both contamination levels and dietary consumption patterns. Strengthening regional food safety efforts is essential, including improved surveillance systems, enhanced post-harvest drying and storage practices, routine monitoring of high-risk commodities, and greater harmonisation of regulatory standards and risk assessment approaches across ASEAN countries. Additionally, the establishment of standardised AFB1 reporting frameworks would improve data comparability and support more robust regional risk assessments.

The present risk estimates were derived from available published consumption data, which may not fully reflect current dietary patterns across the included countries. Nevertheless, our findings may assist policymakers in prioritising interventions targeting high-risk commodities and vulnerable points within regional food supply chains, while addressing current data gaps in underrepresented countries. This study also highlights the need for updated national dietary surveys and improved contamination monitoring across developing ASEAN countries to support more accurate exposure assessment and evidence-based food safety interventions.

## Figures and Tables

**Table 1:** The inclusion and exclusion criteria for study selection.

Inclusion Criteria	Exclusion Criteria
1. Studies on nuts (peanuts/groundnuts) and cereals (rice, maize, wheat) intended for human consumption	1. Studies on non-food items, animal feed, or products not intended for human consumption
2. Studies reporting quantitative data on total aflatoxins (TAF) or AFB1	2. Studies without quantitative AFB1 or TAF data
3. Studies conducted in ASEAN countries	3. Studies conducted outside ASEAN or with non-separable ASEAN data
4. Peer-reviewed primary research articles	4. Secondary literature (reviews, meta-analyses, editorials, conference abstracts)
5. Full-text articles available in English	5. Studies with incomplete reporting (e.g., missing sample size or LO)

**Table 3:** SSW mean AFB1 concentration and uncertainty range (bootstrap) by country and food commodity.

Country	Food	k	Total Samples (n)	AFB1 Concentration ^†^ (µg/kg)	Min ^§^	Max ^§^	Uncertainty Range (Bootstrap) ^‡^ (µg/kg)
Malaysia	Maize	2	13	2.46	0.22	3.86	0.22–3.86
Malaysia	Peanuts	4	285	56.88	4.25	78.55	12.63–70.18
Malaysia	Rice	4	118	5.94	0.95	12.74	1.20–10.29
Philippines	Maize	1	101	489.99	489.99	489.99	NA
Philippines	Peanuts	1	50	2877.26	2877.26	2877.26	NA
Thailand	Peanuts	1	25	1.09	1.09	1.09	NA
Thailand	Rice	3	365	4.72	3.88	11.52	3.88–11.52
Vietnam	Maize	2	567	140.30	74.11	173.40	74.11–173.40
Vietnam	Peanuts	1	144	30.81	30.81	30.81	NA
Vietnam	Rice	2	374	1.46	0.20	3.47	0.20–3.47

^†^ Concentration represents the SSW mean of reported study means. ^§^ Study min and study max represent the minimum and maximum study-level reported mean AFB1 concentrations within each country–commodity group. ^‡^ Uncertainty range obtained by study-level bootstrap (B = 5000) resampling studies with replacement; NA indicates insufficient number of studies (k < 3) to estimate uncertainty using bootstrap resampling.

**Table 4:** Dietary exposure and MOE of AFB1 across selected ASEAN countries.

Country	Food	K ^a^	AFB1 Concentration (µg/kg)			Consumption ^b^ (kg/day)	BW ^b^ (kg)	EDI (ng/kg BW/day)			MOE ^c^		
			Low	Typical	High			Low	Typical	High	Low	Typical	High
Malaysia	Maize	2	0.22	2.46	3.86	0.04	62.65	0.12	1.38	2.17	3236.05	289.40	184.44
Malaysia	Peanuts	4	12.63	56.88	70.18	0.06	62.65	11.47	51.66	63.74	34.87	7.74	6.28
Malaysia	Rice	4	1.19	5.94	10.29	0.25	62.65	4.75	23.70	41.06	84.24	16.88	9.74
Philippines	Maize	1	489.99	489.99	489.99	0.04	63.00	318.88	318.88	318.88	1.25	1.25	1.25
Philippines	Peanuts	1	2877.26	2877.26	2877.26	0.03	63.00	1392.96	1392.96	1392.96	0.29	0.29	0.29
Thailand	Peanuts	1	1.09	1.09	1.09	0	60.00	0.02	0.02	0.02	19,836.35	19,836.35	19,836.35
Thailand	Rice	3	3.88	4.72	11.52	0.14	60.00	8.89	10.81	26.38	45.01	37.00	15.16
Vietnam	Maize	2	74.11	140.30	173.40	0.02	49.00	33.56	63.54	78.53	11.92	6.30	5.09
Vietnam	Peanuts	1	30.81	30.81	30.81	0.01	49.00	9.39	9.39	9.39	42.61	42.61	42.61
Vietnam	Rice	2	0.20	1.46	3.47	0.38	49.00	1.54	11.28	26.80	258.92	35.47	14.92

^a^ For k ≥ 3, uncertainty ranges were estimated using bootstrap resampling; for k = 2, minimum and maximum values are presented; for k = 1, reported values are shown without uncertainty estimation. EDI values are presented under low, typical, and high scenarios as defined in the Methods section. ^b^ Consumption rates and BWs were based on country-specific adult estimates. ^c^ MOE calculated using BMDL_10_ = 400 ng/kg BW/day.

**Table 5:** Estimated liver cancer risk and attributable proportion by country and food commodity under low, typical, and high AFB1 exposure scenarios.

Country	Food	AP	LCR			LCR^AF^ (%)		
			Low	Typical	High	Low	Typical	High
Malaysia	Maize	0.01	0	0.02	0.03	0.76	1.80	2.03
	Peanuts	0.01	0.17	0.76	0.93	70.19	67.31	59.59
	Rice	0.01	0.07	0.35	0.60	29.06	30.89	38.39
Philippines	Maize	0.06	18.63	18.63	18.63	18.63	18.63	18.63
	Peanuts	0.06	81.39	81.39	81.39	81.37	81.37	81.37
Thailand	Peanuts	0.01	0	0	0	0.23	0.19	0.08
	Rice	0.01	0.10	0.12	0.29	99.77	99.81	99.92
Vietnam	Maize	0.03	1.04	1.96	2.42	75.43	75.46	68.45
	Peanuts	0.03	0.29	0.29	0.29	21.10	11.15	8.18
	Rice	0.03	0.05	0.35	0.83	3.47	13.39	23.37

AP: Average potency (cases per year per 100,000 population per ng/kg BW/day). LCR: Liver cancer risk (cases per 100,000 per year). LCR^AF^: Estimated proportion of liver cancer attributable to dietary AFB1 exposure (%).

## Data Availability

The original contributions presented in the study are included in the article/Supplementary Materials; further inquiries can be directed to the corresponding author.

## Supplementary Materials

The following supporting information can be downloaded at: https://www.mdpi.com/article/10.3390/foods15111874/s1. Table S1: Full search strategies for all databases; Table S2: PRISMA-ScR checklist; Table S3: TAF to AFB1 conversion factor; Table S4: Raw AFB1 concentration data with commodity-specific conversion factors applied to TAF-derived values.

## Abbreviations

The following abbreviations are used in this manuscript:

AFB1	Aflatoxin B1
AFB2	Aflatoxin B2
AFG1	Aflatoxin G1
AFG2	Aflatoxin G2
ARAC	ASEAN Risk Assessment Centre for Food Safety
ASEAN	Association of Southeast Asian Nations
TAF	Total Aflatoxins
BMDL_10_	Benchmark Dose Lower-Confidence Limit for a 10% Increase in Response
BW	Body Weight
CR	Consumption Rate
EDI	Estimated Daily Intake
ELISA	Enzyme-Linked Immunosorbent Assay
EU	European Union
FAO	Food and Agriculture Organisation
HBsAg	Hepatitis B Surface Antigen
HBV	Hepatitis B Virus
HCC	Hepatocellular Carcinoma
HPLC	High-Performance Liquid Chromatography
IAC	Immunoaffinity Column
IARC	International Agency for Research on Cancer
LC–ESI-QTOF-MS/MS	Liquid Chromatography–Electrospray Ionisation–Quadrupole Time-of-Flight–Tandem Mass Spectrometry
LC–MS/MS	Liquid Chromatography–Tandem Mass Spectrometry
LOD	Limit of Detection
MANS	Malaysian Adult Nutrition Survey
ML	Maximum Level
MOE	Margin of Exposure
PCC	Population–Concept–Context
PRISMA-ScR	Preferred Reporting Items for Systematic Reviews and Meta-Analyses Extension for Scoping Reviews
PROSPERO	International Prospective Register of Systematic Reviews
SD	Standard Deviation
SSW	Sample-Size–Weighted
UPLC-FLD	Ultra-Performance Liquid Chromatography with Fluorescence Detection
WHO	World Health Organization
