# Supplementary material for: A Scoping Review and Risk Assessment of Aflatoxin B1 Contamination in Rice, Maize, and Peanut-Based Products Across Selected ASEAN Countries
Source: Foods. 2026 May 25;15(11):1874. doi: 10.3390/foods15111874 (PMC13256838; doi:10.3390/foods15111874)
Supplement: Supplementary file 1 [file foods-15-01874-s001.zip › Supplementary Table S1.pdf]

Supplementary Table S1: Full search strategies for all databases

Database	Search strings
Pubmed	‘aflatoxin*’ OR ‘afb1’ OR 'aflatoxin b1'<br>‘maize’ OR ‘rice' OR ‘corn’ OR ‘peanut’ OR ‘nut’ OR 'groundnut'<br>(‘aflatoxin*’ OR ‘afb1’ OR 'aflatoxin b1') AND (‘maize’ OR ‘rice' OR ‘corn’ OR ‘peanut’ OR ‘nut’ OR 'groundnut')<br>(‘aflatoxin*’ OR ‘afb1’ OR 'aflatoxin b1’) AND (‘maize’ OR ‘rice' OR ‘corn’ OR ‘peanut’ OR ‘nut’ OR 'groundnut') Filters: from 2010 - 2025
Scopus	‘maize’ OR ‘rice' OR ‘corn’ OR ‘peanut’ OR ‘nut’ OR 'groundnut'<br>‘aflatoxin*’ OR ‘afb1’ OR 'aflatoxin b1'<br>(‘aflatoxin*’ OR ‘afb1’ OR 'aflatoxin b1') AND (‘maize’ OR ‘rice' OR ‘corn’ OR ‘peanut’ OR ‘nut’ OR 'groundnut')<br>‘aflatoxin*’ OR ‘afb1’ OR 'aflatoxin b1' AND (‘maize’ OR ‘rice' OR ‘corn’ OR ‘peanut’ OR ‘nut’ OR 'groundnut') Filters: from 2010 - 2025
Web of Science	aflatoxin* (All Fields) or afb1 (All Fields) or aflatoxin b1 (All Fields)<br>maize (All Fields) or rice (All Fields) or corn (All Fields) or peanut (All Fields) or nut (All Fields) or groundnut (All Fields)<br>“aflatoxin* (All Fields) or afb1 (All Fields) or aflatoxin b1 (All Fields) AND “maize (All Fields) or rice (All Fields) or corn (All Fields) or peanut (All Fields) or nut (All Fields) or groundnut (All Fields)”
Embase	‘aflatoxin*’:ti,ab,kw OR ‘afb1’:ti,ab,kw OR 'aflatoxin b1':ti,ab,kw<br>maize:ti,ab,kw OR rice:ti,ab,kw OR corn:ti,ab,kw OR peanut:ti,ab,kw OR nut:ti,ab,kw OR groundnut:ti,ab,kw

	“‘aflatoxin*’:ti,ab,kw OR ‘afb1’:ti,ab,kw OR 'aflatoxin b1':ti,ab,kwAND “maize:ti,ab,kw OR rice:ti,ab,kw OR corn:ti,ab,kw OR peanut:ti,ab,kw OR nut:ti,ab,kw OR groundnut:ti,ab,kw”
Wiley	"'aflatoxin b1' OR AFB1 OR aflatoxin" in Abstract AND "maize OR rice OR corn OR peanut OR nut OR groundnut" in Abstract
