# Supplementary material for: A Scoping Review and Risk Assessment of Aflatoxin B1 Contamination in Rice, Maize, and Peanut-Based Products Across Selected ASEAN Countries
Source: Foods. 2026 May 25;15(11):1874. doi: 10.3390/foods15111874 (PMC13256838; doi:10.3390/foods15111874)
Supplement: Supplementary file 1 [file foods-15-01874-s001.zip › Supplementary Table S3.pdf]

Supplementary Table S3

Table A: AFT to AFB1 Conversion Factor for rice.

Mean AFB1 (µg/kg)	Mean AFT (µg/kg)	AFB1 (% AFT)	Correction factor AFB1→AFT (100/AFB1)	Correction factor AFT→AFB1 (AFB1/100)
3.56	7.61	46.78	2.14	0.47
1.21	3.5	34.57	2.89	0.35
1.21	3.87	31.27	3.20	0.31
2.42	3.55	68.17	1.47	0.68
Average		45.20	2.42	0.45
Std Dev		16.71	0.78	0.17

Source: Khalisanni K, Napiah N, Ishak R, Siva R, Abdullah S. Occurrences of Aflatoxins in Selected Rice Production Areas in Malaysia. J Trop Plant Physiol. 2022 Jun 1;14:8.

Table B: AFT to AFB1 Conversion Factor for peanut.

Mean AFB1 (µg/kg)	Mean AFT (µg/kg)	AFB1 (% AFT)	Correction factor AFB1→AFT (100/AFB1)	Correction factor AFT→AFB1 (AFB1/100)
60.67	72.94	83.18	1.2	0.83
2.95	4.4	67.05	1.49	0.67
3.04	7.56	40.21	2.49	0.4
7.93	9.51	83.39	1.2	0.83
2.87	2.87	100	1	1
87.02	97.28	89.45	1.12	0.89
2.76	2.76	100	1	1
2.89	2.89	100	1	1
4.81	7.67	62.71	1.59	0.63
6.46	9.15	70.6	1.42	0.71
6.24	8.7	71.72	1.39	0.72
7.72	9.53	81.01	1.23	0.81
2.86	3.81	75.07	1.33	0.75
2.72	3.7	73.51	1.36	0.74
7.84	10.25	76.49	1.31	0.76
8.27	10.98	75.32	1.33	0.75

17.81	21.79	81.73	1.22	0.82
2.8	3.78	74.07	1.35	0.74
4.17	9.75	42.77	2.34	0.43
2.92	3.91	74.68	1.34	0.75
7.14	9.07	78.72	1.27	0.79
Average			1.38	0.763
Std Dev			0.377	0.155

Source: Arzandeh S, Selamat J, Lioe H. Aflatoxin in raw peanut kernels marketed in Malaysia. *J Food Drug Anal.* 2010;18(1):44–50.

Table C: AFT to AFB1 Conversion Factor for corn.

Mean AFB1 (µg/kg)	Mean AFT (µg/kg)	AFB1 (% AFT)	Correction factor AFB1→AFT (100/AFB1)	Correction factor AFT→AFB1 (AFB1/100)
30	30	100	1	1
46	62	74.19	1.35	0.74
92	99	92.93	1.08	0.93
49	58	84.48	1.18	0.84
40	48	83.33	1.2	0.83
39	39	100	1	1
90	111	81.08	1.23	0.81
4	5	80	1.25	0.8
252	301	83.72	1.19	0.84
428	487	87.89	1.14	0.88
Average			1.162	0.868
Std Dev			0.111	0.085

Source: Dharmaputra OS. Fungi, mycotoxins and their control in Indonesian food and feedstuff. Bogor (Indonesia): *Seameo Biotrop.* 2014.

Table D: AFB1 Contamination Levels in Peanuts, Maize, and Rice Across ASEAN Countries.

Country	Reference	Food type	n	Toxin	Mean	SD	Min	Max	Median
Philippines	Romero & Cumagun (2023)	Maize	5	AFB1	14.5824			12	
Philippines	Romero & Cumagun (2023)	Maize	5	AFB1	11.0236				
Philippines	Romero & Cumagun (2023)	Maize	18	AFB1	40.6224				
Philippines	Romero & Cumagun (2023)	Maize	21	AFB1	193.7376				
Philippines	Romero & Cumagun (2023)	Maize	22	AFB1	89.0568				
Philippines	Romero & Cumagun (2023)	Maize	30	AFB1	140.9632				

Philippines	Rustia et al. (2022)	Peanut	12	AFB1	30.5	54.02			
Philippines	Rustia et al. (2022)	Peanut	12	AFB1	2337.04	3463.24			
Philippines	Rustia et al. (2022)	Peanut	12	AFB1	465.5	566.22			
Philippines	Rustia et al. (2022)	Peanut	10	AFB1	0.51	0.2			
Philippines	Rustia et al. (2022)	Peanut	4	AFB1	43.71	78.62			
Thailand	Panrapee et al. (2016)	Coloured Rice	30	AFB1	1.66			26.61	
Thailand	Panrapee et al. (2016)	Coloured Rice	30	AFB1	0.76			13.42	
Thailand	Panrapee et al. (2016)	Coloured Rice	30	AFB1	0.57			5.8	
Thailand	Panrapee et al. (2016)	Coloured Rice	30	AFB1	0.41			4.16	
Thailand	Panrapee et al. (2016)	Coloured Rice	30	AFB1	0.09			2.61	
Thailand	Panrapee et al. (2016)	Coloured Rice	30	AFB1	0.05			1.43	
Thailand	Panrapee et al. (2016)	Coloured Rice	30	AFB1	0.14			2.02	
Thailand	Panrapee et al. (2016)	Coloured Rice	30	AFB1	0.2			3.51	
Thailand	Sinphitakkul et al. (2019)	Coloured Rice	30	AFB1	1.38			0.4	
Thailand	Sinphitakkul et al. (2019)	Coloured Rice	30	AFB1	1.44			0.5	
Thailand	Sinphitakkul et al. (2019)	Coloured Rice	30	AFB1	1.48			0.4	
Thailand	Tansakul et al. (2013)	Rice	25	AFB1	0.072				
Thailand	Tansakul et al. (2013)	Rice	10	AFB1	11.4435			50.79	
Thailand	Tansakul et al. (2013)	Peanut	25	AFB1	1.09109			7.39	
Vietnam	Nguyen et al. (2018)	Maize	76	AFB1	73.7			417	41
Vietnam	Nguyen et al. (2018)	Maize	75	AFB1	16			66.8	4.5
Vietnam	Nguyen et al. (2018)	Maize	76	AFB1	45.8			141.8	34.3
Vietnam	Nguyen et al. (2018)	Maize	76	AFB1	26.6			55.6	26.7
Vietnam	Nguyen et al. (2018)	Maize	75	AFB1	11.3			69.4	4.6
Vietnam	Phan et al. (2021)	Rice	230	AFB1	0.2	0.4			10.2
Vietnam	Huong et al. (2019)	Rice Product	3	AFB1				2.4	3.02
Vietnam	Do et al. (2020)	Maize	40	AFB1	2.62			25	
Vietnam	Do et al. (2020)	Rice	40	AFB1	0.42			13	
Vietnam	Do et al. (2020)	Peanut	40	AFB1	9.28			16	
Vietnam	Do et al. (2020)	Maize	49	AFB1	5.39			94.5	
Vietnam	Do et al. (2020)	Rice	48	AFB1	2.04			93	
Vietnam	Do et al. (2020)	Peanut	46	AFB1	4.96			159	
Vietnam	Do et al. (2020)	Maize	100	AFB1	66.1			1572	
Vietnam	Do et al. (2020)	Rice	56	AFB1	1.01			26	

Vietnam	Do et al. (2020)	Peanut	58	AFB1	16.57			362	
Malaysia	Chin et al. (2012)	Peanut		AFB1	26.75		ND	117.3	
Malaysia	Samsudin & Abdullah (2013)	Coloured Rice	50	AFB1	6.624	16.24		77.33	
Malaysia	Norlia et al. (2018)	Peanut	87	AFB1	56	183.4		995.4	0
Malaysia	Norlia et al. (2018)	Peanut Product	91	AFB1	6	24.4		196.7	0.2
Malaysia	Soleimany et al., (2011)	Rice	30	AFB1	0.954	0.26		3.96	
Malaysia	Soleimany et al., (2011)	Maize	5	AFB1	0.217				
Malaysia	Sirhan et al. (2013)	Peanut		AFB1	23				
Malaysia	Khayoon et al. (2012)	Coloured Rice	5	AFB1	2.349				
Malaysia	Khayoon et al. (2012)	Coloured Rice	5	AFB1	0.495				
Malaysia	Khayoon et al. (2012)	Rice	5	AFB1	2.2365				
Malaysia	Khayoon et al. (2012)	Rice	5	AFB1	2.376				
Malaysia	Khayoon et al. (2012)	Rice	5	AFB1	5.28				
Malaysia	Khayoon et al. (2012)	Peanut	9	AFB1	6				
Malaysia	Khayoon et al. (2012)	Peanut	9	AFB1	1.64045				
Malaysia	Khayoon et al. (2012)	Peanut	9	AFB1	3.16				
Malaysia	Khayoon et al. (2012)	Peanut	9	AFB1	4.85268				
Malaysia	Reddy et al. (2011)	Rice Product	13	AFB1	1.75			3.79	
Malaysia	Reddy et al. (2011)	Corn Product	8	AFB1	3.86			8.95	
Malaysia	Reddy et al. (2011)	Peanut	13	AFB1	4.25			15.33	
Malaysia	Leong et al., (2011)	Peanut Product	8	AFB1	1.01	0.02		1.02	
Malaysia	Leong et al., (2011)	Peanut Product	8	AFB1	0.78	0.43		1.08	
Malaysia	Leong et al., (2011)	Peanut Product	5	AFB1	58.9	93.4		222	
Malaysia	Leong et al., (2011)	Peanut Product	2	AFB1	0.68	0.39		0.96	
Malaysia	Leong et al., (2011)	Peanut Product	19	AFB1	5.2	11		44.1	
Malaysia	Leong et al., (2011)	Peanut Product	4	AFB1	1.19	0.18		1.48	
Malaysia	Leong et al., (2011)	Peanut Product	4	AFB1	2.77	2.11		5.98	
Malaysia	Leong et al., (2011)	Peanut Product	3	AFB1	6.03	3.24		10.2	
Malaysia	Leong et al., (2011)	Peanut Product	3	AFB1	0.99	0.15		1.18	
Malaysia	Leong et al., (2011)	Peanut Product	2	AFB1	1	0.02		1.02	
Vietnam	Nguyen et al., (2023)	Peanut Product	350	AFB1				554	
